# The effects of forest conversion to oil palm on ground-foraging ant communities depend on beta diversity and sampling grain

**DOI:** 10.1002/ece3.1592

**Published:** 2015-07-14

**Authors:** Wendy Y Wang, William A Foster

**Affiliations:** Department of Zoology, University of CambridgeCambridge, U.K

**Keywords:** Agriculture, converted landscapes, Hill numbers, insect diversity, multi-assemblage similarities, scale

## Abstract

Beta diversity – the variation in species composition among spatially discrete communities – and sampling grain – the size of samples being compared – may alter our perspectives of diversity within and between landscapes before and after agricultural conversion. Such assumptions are usually based on point comparisons, which do not accurately capture actual differences in total diversity. Beta diversity is often not rigorously examined. We investigated the beta diversity of ground-foraging ant communities in fragmented oil palm and forest landscapes in Sabah, Malaysia, using diversity metrics transformed from Hill number equivalents to remove dependences on alpha diversity. We compared the beta diversities of oil palm and forest, across three hierarchically nested sampling grains. We found that oil palm and forest communities had a greater percentage of total shared species when larger samples were compared. Across all grains and disregarding relative abundances, there was higher beta diversity of all species among forest communities. However, there were higher beta diversities of common and very abundant (dominant) species in oil palm as compared to forests. Differences in beta diversities between oil palm and forest were greatest at the largest sampling grain. Larger sampling grains in oil palm may generate bigger species pools, increasing the probability of shared species with forest samples. Greater beta diversity of all species in forest may be attributed to rare species. Oil palm communities may be more heterogeneous in common and dominant species because of variable community assembly events. Rare and also common species are better captured at larger grains, boosting differences in beta diversity between larger samples of forest and oil palm communities. Although agricultural landscapes support a lower total diversity than natural forests, diversity especially of abundant species is still important for maintaining ecosystem stability. Diversity in agricultural landscapes may be greater than expected when beta diversity is accounted for at large spatial scales.

## Introduction

The conversion of tropical forest landscapes to agriculture is a major factor driving the global biodiversity crisis (Sodhi et al. 2010). Biodiversity worldwide is increasingly being fractionated into landscape matrices characterized by mosaics of pristine forest and agricultural lands. In such fragmented ecosystems, multiple factors may alter assemblages occupying different tracts of the mosaic. To date, however, our understanding of the impacts of agricultural conversion on native diversity is mostly informed by studies limited to point comparisons between conversion and preconversion habitats. Beta diversity – the variation in species composition among spatially discrete communities (Whittaker 1972; Flohre et al. 2011) – and the spatial scale of sampling are not usually rigorously examined. Consequently, such studies may have distorted and over-simplified the more nuanced effects of agricultural conversion on native diversity. Beta diversity and its underlying processes in landscapes fragmented by agriculture are hence not well understood, especially in the tropics (Karp et al. 2012; Barton et al. 2013).

A comprehensive understanding of beta diversity is critical toward the conservation and sustenance of species diversity in fragmented landscapes. Beta diversity patterns may vary at different scales, driven by ecological or physical phenomena operating at either local or larger regional levels (Flohre et al. 2011; Steinbauer et al. 2012). In particular, beta diversity can depend on sampling grain: the smallest unit of comparison in a study which defines the lower limit of data resolution (Wiens 1989). At small grains, beta diversity may be shaped by biological interactions between species occurring at local scales (Laliberté et al. 2009). In contrast, beta diversity at large grains may be strongly predicted by landscape components such as landscape diversity (Fahrig et al. 2011) and matrix composition (Bennett et al. 2006). Species associations with vegetation and climatic factors are also more prevalent at broad spatial scales (Laliberté et al. 2009).

Landscapes converted to agriculture are often assumed to be structurally more homogeneous than natural preconversion landscapes, consequently supporting a less diverse fauna (Tscharntke et al. 2005). Empirical information from existing studies, however, tends to be divergent on the effects of modification and fragmentation on beta diversity at different scales. Karp et al. (2012) showed that beta diversity of tropical bird communities in Costa Rica decreased in landscapes homogenized by high-intensity agriculture at large spatial scales. In contrast, Hill and Hamer (2004) found that the beta diversity of tropical birds in habitats disturbed by logging or shifting agriculture was more likely to be higher at larger spatial scales. Further, Arroyo-Rodríguez et al. (2013) showed that the beta diversity of tropical plant communities was lower in severely deforested landscapes, relative to landscapes with less deforestation, but only at the smallest spatial scale. Moreover, undisturbed habitats do not necessarily support greater diversity. For example, Novotny et al. (2007) found low beta diversity of herbivorous insects in contiguous rainforests of Papua New Guinea, suggesting a more homogeneous natural forest fauna than is usually expected. It is evident from these studies that diversity in fragmented landscapes is contingent on beta diversity and the scale at which communities are assessed.

The main aim of our study was to establish whether a rigorous consideration of beta diversity, and its relationship to sampling grain would affect our understanding of the impacts of converting natural habitats to agriculture on biodiversity in the tropics. We chose to study ants in oil palm (*Elaeis guineensis*), because this crop dominates agricultural ecosystems in Indonesia and Malaysia and is also widespread in many other tropical regions; it is a major driver of forest loss wherever it is grown (Fitzherbert et al. 2008). Conversion of forest to oil palm drastically reduces species diversity across most taxonomic groups (Foster et al. 2011).

Our study focuses on ants, one of the most prominent taxa in terrestrial landscapes worldwide, undertaking functionally important roles in many ecosystems (Hölldobler and Wilson 1990). They are ubiquitous in oil palm, but the oil palm ant fauna is currently considered impoverished in comparison with that of native forests, based on point counts at local scales (Brühl and Eltz 2010; Fayle et al. 2010). Ants usually form stationary and perennial nests, and have fairly restricted foraging ranges (Alonso 2000), ensuring that those occurring in a sample are not merely transient tourists, but species actually inhabiting a particular site. Despite their ubiquity, there is limited understanding of the beta diversity of ants in tropical ecosystems (Pfeiffer and Mezger 2012; Rizali et al. 2013), particularly for landscapes fragmented by agriculture such as oil palm.

The beta diversity of ants in any landscape may be expected to fall with habitat homogenization, which is often assumed to occur as an inevitable consequence of forest conversion to agriculture. More homogenized habitats entail a lower diversity of resources available, potentially leading to species declines especially for specialists that thrive only on particular resources (Fayle et al. 2010). A small number of wide-ranging generalist species may dominate communities in such agricultural landscapes. Alternatively, ant communities in converted landscapes may exhibit greater compositional variation, considering that these communities are usually at different stages of succession in species assembly, and are hence subject to varied influences such as the colonization sequence of species from the surrounding matrix (Fukami 2010). Furthermore, there might be more structural heterogeneity in agricultural landscapes than assumed, such as for perennial crops with mixed age stands (Foster et al. 2011), possibly supporting a wider range of species with different habitat requirements.

As oil palm is a monoculture, both the oil palm landscape and its associated animal and plant communities might be assumed to be relatively homogeneous, as compared to a forest landscape (Tscharntke et al. 2005). If this were the case, point comparisons at local scales, which would not account for the higher beta diversity in forest compared with oil palm, might seriously underestimate the effects of forest conversion on biodiversity. We therefore compared the beta diversity of ground-foraging ants within and between oil palm and forest landscapes in Sabah (Malaysia), adopting a multiscale approach with three hierarchically nested sampling grains. Our main objective was to test whether oil palm ant communities were more homogenized than forest communities, as expected of converted landscapes, and whether results were comparable at different scales of sampling. We sampled the ground-foraging ant fauna using unbaited pitfall traps, which may not give a complete and accurate census of all species in each community, but still provides a good representation of ground surface-active ants in an area (Bestelmeyer et al. 2000).

Methods used by past studies to compare compositional differences between spatially discrete communities are usually dependent on alpha diversity or the observed number of species per locality (Chao et al. 2012). Beta diversity might be inflated by a lower alpha diversity of each community sampled in a particular region (Karp et al. 2012). We removed this dependency using transformed multiple-community generalizations of pairwise similarity measures (Chao et al. 2012), allowing for fairer comparisons of beta diversity between tropical oil palm, which is expected to have lower total diversity, and forest landscapes. We are concerned with general beta diversity, regardless of identities and perceived conservation values of individual species, in this study. Species labeled as “exotics” or “tramps” of recognized low conservation value may, nevertheless, play important roles in maintaining well-functioning ecosystems (Prévot-Julliard et al. 2011). Beta diversity of all species is therefore linked to ecosystem sustainability, also a major component of conservation.

## Materials and methods

### Study sites

**Figure 1:**
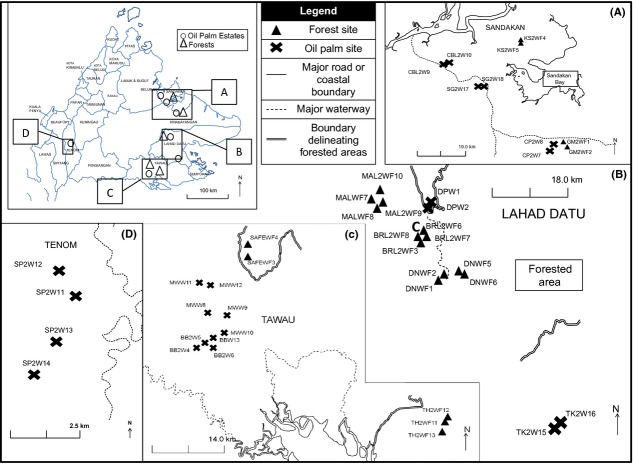
Locations of 26 oil palm and 21 forest sites sampled in Sabah, Malaysia. Oil palm estate names: BB – Bukit Batu (Sabah Softwoods Bhd), CBL – Cepatwawasan Beluran, CP – Cepatwawasan, DP – Danum Palm, MW – Mawang (Sabah Softwoods Bhd), SG – Segaliud (Sime Darby), SP – Sapong (Sime Darby), TK – Tingkayu (Sime Darby). Forest names: DN – Danum Valley Conservation Area, BRL – Borneo Rainforest Lodge Forest Reserve, GM – Gomantong, KS – Kabili-Sepilok, MAL – Ulu Segama-Malua, SAFE – Stability of Altered Forest Ecosystems Project Virgin Jungle Reserve, TH – Tawau Hills Park. Starting letters indicate the oil palm estate (_W) or forest (_WF) where the plot is located. Letters followed by the number “2” indicate plots sampled in 2012; 2011 otherwise.

We sampled a total of 26 oil palm plots and 21 forest sites across Sabah, Malaysia (Fig.1). Sabah has a land area of 73,631 km^2^, taking up approximately 10 percent of Borneo, East Malaysia (Reynolds et al. 2011). Its overall landscape comprises a fragmented mosaic of multiple land use and forest types, whereby oil palm plantations occupy more than 14,000 km^2^ or about 19 percent of total land area (Reynolds et al. 2011). Oil palm within the age range of 8–13 years was chosen for sampling as this corresponded roughly to the period of steadily rising yield in the palm oil production cycle (Ismail and Mamat 2002). Forest sites sampled consisted of primary and secondary lowland dipterocarp forests corresponding to different classes of the Sabah Forestry Department’s classification system (Reynolds et al. 2011) (Fig.1). Oil palm plots were positioned at least 1 km away from forested areas or fragments to avoid spillover effects on ant species; forest plots were also located at least 500 m from roads or man-made boundaries to avoid potential edge effects in sampling. Sampling was conducted in April–September 2011 and 2012.

### Derivation of sampling grains

Variation among ant assemblages was analyzed at three hierarchical nested grains, obtained by pooling pairs of either oil palm or forest samples of the smaller grain to the next larger grain, while total sampled area remained fixed. A 100 × 100 m^2^ plot was taken as the first and also smallest sampling grain (Grain 1: *N*_oil palm_ = 26, *N*_forest_ = 21). Samples from paired plots in relatively close proximity (oil palm: 1.12–4.08 km; forest: 1.17–2.27 km) were aggregated to form the second level of the hierarchy (Grain 2: *N*_oil palm_ = 13, *N*_forest_ = 10). One forest plot was omitted from the pairings, as we could not access and sample another plot within close vicinity (∼5 km) to form a separate pair. The third, and largest, grain was obtained by further pooling pairs of samples of the second grain (Grain 3: *N*_oil palm_ = 6, *N*_forest_ = 5). A pair of oil palm plots were omitted from this round of sample pooling, because there were inadequate plots to form nonoverlapping grains comprising four plots each. Samples pooled together at the largest grain were from plot-pairs distributed within 6–45 km of each other.

### Field methods

We installed 30 unbaited pitfall traps in each 100 m^2^ plot, positioned around five random focal trees spaced at least 20 m apart. Four traps were installed in a North–South–East–West configuration five meters from each focal tree, and two more traps were installed approximately one meter from the tree base. Each trap comprised of a 50-mL centrifuge tube (Falcon™) filled with 15 mL 95% denatured ethanol and a few drops of unscented detergent. Traps were collected after 48 h and sorted for ants thereafter. Unbaited pitfall traps capture a good representation of most ants that forage on the ground (Bestelmeyer et al. 2000) and their relative abundances (Gotelli et al. 2011; Stuble et al. 2013). Using unbaited pitfall traps is therefore sufficient for the main purposes of this study, ensuring standardized sampling effort in both oil palm and forest, allowing for fair comparisons of samples between the two habitat types.

### Ant identification and biomass estimation

Ants collected from pitfall traps were counted and identified to morphospecies, using references and available taxonomic keys (e.g., Hashimoto 2003). Ant biomass was used as a surrogate for abundance in terms of individual counts for subsequent calculation of diversity metrics, in order to account for broad variation in size both within and among species. Furthermore, the proportional biomass representation of a species within a community usually reflects its potential influence on ecosystem processes, regardless of individual size. We therefore chose to use biomass instead of individual counts in view of its possible implications on ecosystem function. Mean biomass for individuals of each morphospecies collected from pitfall traps was calculated from weights of at least three oven-dried specimens per species and then used to convert abundance counts to biomass for all samples. Mean biomasses of major and minor castes were calculated separately. Regression curves of mean dry weight against various body dimensions measured from sampled ants were used to predict mean individual biomass for species represented by less than three intact specimens. Following Kaspari and Weiser (1999), we measured different body dimensions for different subfamilies: headlength for Dolichoderines, Formicines and Pseudomyrmicines, tibia length for Ponerines, and pronotum width for Myrmicines.

### Assessing sampling completeness and species ranked abundances

Sample-based species and coverage-based rarefaction curves were generated for oil palm and forest samples, respectively, to assess sampling completeness. (Appendix S1). Coverage refers to the proportion of the total abundance of individuals in an assemblage that belongs to species represented in the sample (Chao et al. 2014). Rarefied species richness was plotted against the number of samples and coverage, respectively. Further, to examine how sampling completeness varied with the number of samples, sampling coverage was plotted as a function of number of samples for oil palm and forest, respectively. Sampling curves were constructed using the online freeware application iNEXT (iNterpolation/EXTrapolation) (Hsieh et al. 2013).

Bar graphs illustrating the top 20 species, in biomass (mg) per unit of occurrence (grain) for each grain level, were constructed to visualize possible shifts in species ranks at different grains. These graphs were adapted from standard rank–abundance curves, using biomass per grain as a surrogate for individual counts.

### Data analyses

#### Alpha diversity and average pairwise overlap between oil palm and forest communities

Average true alpha diversities (^*q*^*D*_*α*_) were first calculated for each grain using Hill numbers (Hill 1973), which represent the “effective number of species within a community” (Jost 2007). Different values of *q* in each measure give differential weights to the relative abundances of species (Jost et al. 2011). The measure ^0^*D*_*α*_ corresponds to species richness, ^1^*D*_*α*_ approximates the number of common species, and ^2^*D*_*α*_ represents the number of very abundant or dominant species in a community (Chao et al. 2014). We are interested in investigating the diversities of common and dominant species, because by virtue of their relative abundances, these species potentially play important functional roles in the ecosystems they inhabit, regardless of species identities.

^*q*^*D*_*α*_ can be calculated as follows:

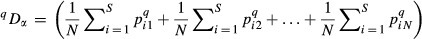
1*S* denotes the total number of species found across all *N* assemblages being compared, and *p*_*i*_ represents the relative abundance, surrogated with proportional biomass in this case, of the *i*-th species.

To evaluate average similarity between an oil palm and a forest community, all possible pairwise combinations of between-group (oil palm against forest) comparisons were generated at each grain. Pairwise overlap was assessed using three similarity indices: Sørensen’s index measuring similarities in species identities, the Horn index for similarities in common species, and the Morisita–Horn index for similarities in dominant species. Standard error (SE) for each similarity value was obtained through bootstrapping at 500 iterations. Mean pairwise similarity was then calculated from the set of comparisons at each grain and value of *q*. Differences between similarity values at different grains were assessed using standardized *Z* tests.

#### Comparisons of beta diversity within and between oil palm and forest landscapes

The family of overlap measures (C_*qN*_) includes multiple-assemblage generalizations of classic pairwise similarity indices (Jost et al. 2011). These similarity measures are derived from traditional beta metrics which have been transformed to remove dependences on alpha diversity and the number of communities, allowing for fair comparisons of relative similarity among communities across multiple regions (Chao et al. 2012). C_*qN*_ can be interpreted as the percentage species overlap in a set of *N* equivalent communities, where *q* determines sensitivity of the measure to species’ relative frequencies (Chao et al. 2014):

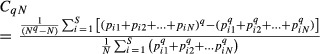
2when *q* = 0, the multi-assemblage Sørensen’s index is obtained:

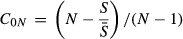
3*S* denotes the total number of species across *N* equally weighted communities, whereas 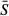 represents the mean number of species per assemblage.

C_1*N*_ gives the generalized Horn (1966) index of overlap as *q* tends to 1:

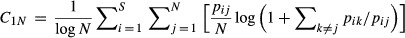
4

C_2*N*_ gives the generalized multi-assemblage Morisita–Horn index, when *q* = 2:

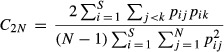
5

C_*qN*_ values between the three sampling grains, and between oil palm and forest at each grain, were compared using standardized *Z* tests. Standard error values for each parameter output were derived from bootstrapping at 1000 random iterations of each original dataset.

Significances of all test statistics from comparisons between grains were corrected for multiple testing of grain levels using a simple sequential Bonferroni-type false discovery rate (FDR) procedure (Benjamini and Hochberg 1995). Singletons were included in the main round of data analyses, but later omitted for a repeated set of analyses to determine whether very rare species affected assessments of beta diversity.

For ease of explanation, results of comparisons with C_*qN*_ are reported as changes to beta diversity (^*q*^*β*_grain1/2/3_) for either oil palm or forest communities, at different orders of *q*. ^*q*^*β* can be obtained simply by subtracting C_*qN*_ from 1, that is, ^*q*^*β* = 1−C_*qN*_.

All measures of diversity and matrices were generated, and statistical tests performed in R ver. 2.14.1 (R Development Core Team 2011), with packages *vegetarian* (Charney and Record 2010) and *Vegan* (Oksanen et al. 2011).

## Results

### General species overview

We collected a total of 223 morphospecies of ground-foraging ants from 10 subfamilies and 58 genera (Table S1). From oil palm obtained 14,525 individuals (5992.4 mg) from 105 species, while 11,501 individuals (8401.4 mg) from 181 species were identified from forest samples. Sixty-three species were shared between oil palm and forest, which means 40 percent of oil palm species were exclusive to oil palm, and approximately 65.2 percent of forest species were only found in forest. Rarefied coverage accumulation curves indicated that coverage for <26 samples was slightly higher in oil palm than forest, that is for the same level of coverage, more forest samples are required (Appendix S1). While our samples were probably incomplete representations of the total ground-foraging ant fauna per habitat, confidence intervals for coverage curves of both oil palm and forest overlap (Appendix S1), suggesting that comparisons of diversity between these two landscapes will not be confounded by biased sampling completeness.

Mean effective species richness (^0^*D*_*α*_) and mean effective numbers of common species (^1^*D*_*α*_) and dominant species (^2^*D*_*α*_) were significantly higher (*P* < 0.05) in forest than in oil palm at all three grains (Table1). Four species were consistently ranked among the top 10 in terms of mean biomass per sampling unit (grain) across all three grains in both oil palm and forest, namely *Leptogenys mutabilis, Lophomyrmex bedoti, Odontoponera transversa*, and *Pheidologeton affinis* (Appendix S2). Various species changed in ranking by biomass with changes in sampling grain size (Appendix S2).

**Table 1:** Summary of mean alpha diversity in oil palm and forest communities at different grains

Grain	*N*		0*D*_*α*_ [sp richness]		1*D*_*α*_ [common spp]		2*D*_*α*_ [dominant spp]	
	Oil palm	Forest	Oil palm	Forest	Oil palm	Forest	Oil palm	Forest
1	26	21	18.5 (0.28)	31.8 (0.42)	2.8 (0.06)	4.5 (0.08)	1.8 (0.03)	2.5 (0.04)
2	13	10	28.6 (0.48)	49.9 (0.76)	3.4 (0.06)	5.5 (0.12)	2.1 (0.03)	2.7 (0.05)
3	6	5	43.8 (0.90)	77.2 (1.33)	3.8 (0.08)	7.1 (0.16)	2.3 (0.03)	3.4 (0.07)

*N* – number of samples/communities used to calculate the mean.

0 *D*_*α*_ – Mean effective species richness

1 *D*_*α*_ – Mean effective number of common species

2 *D*_*α*_ – Mean effective number of dominant species. Standard error (S.E) values are displayed in parentheses. All values of ^*q*^*D*_*α*_ from forest displayed are significantly (*P* < 0.05) higher than those from oil palm.

### Average pairwise similarities between oil palm and forest

**Figure 2:**
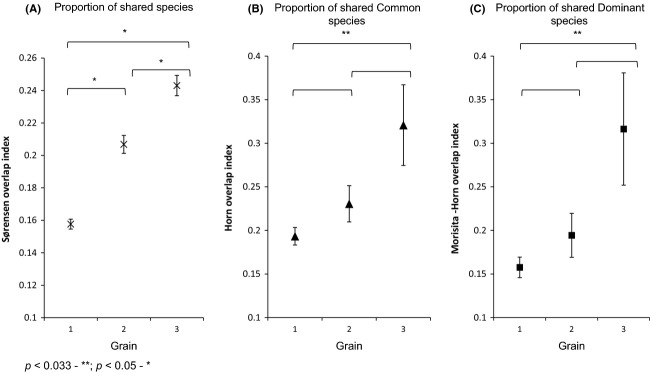
Average pairwise species comparisons between oil palm and forest ground-foraging ant assemblages across three grains (1–3). Sørensen overlap index – proportion of shared species (all, including rare species); Horn overlap index – proportion of shared common species; Morisita–Horn index – proportion of shared very abundant/dominant species. Error bars depict standard errors of each mean similarity computed from all possible oil palm – forest pairs per grain. Statistically significant *Z* test comparisons between grains are marked with **(*P *< 0.033), or *(*P* < 0.05). Significance levels have been FDR-corrected using a sequential Bonferroni procedure.

Sampling grain had a marked effect on the apparent percentage overlap of species between a forest and an oil palm community. Average pairwise similarities in effective species identities (Sørensen’s index), common species (Horn index), and dominant species (Morisita–Horn index) between an oil palm and a forest community were highest at the third grain, relative to the first grain (Fig.2). Average similarity in species identities significantly increased (*P* < 0.05) progressively from the first grain to the second and from the second grain to the third. Average similarities between an oil palm and a forest community were significantly higher at the third grain as compared to the first for both common and dominant species (Fig.2). However, average similarities at the second grain were not significantly different from the first grain for common and dominant species; similarities at the second grain were also not significantly different from similarities at the third grain, respectively (Fig.2).

### Patterns of beta diversity within oil palm and forest across different grains

“Beta diversity” in this section refers to the metric ^*q*^*β*, which is derived from 1−C_*qN*_ (see Materials and methods). C_*qN*_ may be interpreted as the percentage species overlap in a set of *N* equivalent communities, of dominant species (*q* = 2), common species (*q* = 1), or species identities disregarding relative abundances (*q* = 0).

**Figure 3:**
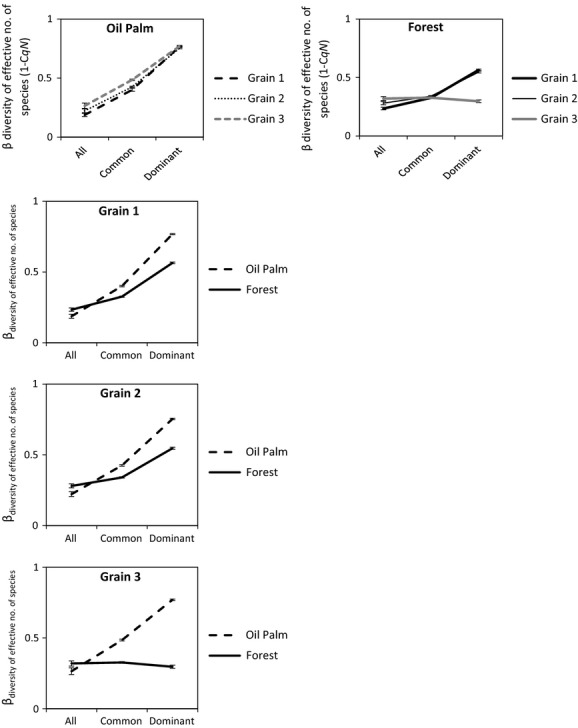
Comparisons of beta diversity (^q^*β*, or 1−C_*qN*_) within and between oil palm and forest landscapes at three grains (1–3) and for (*x*-axis): (1) all species (*q* = 0), (2) common species (*q *= 1) and (3) very abundant/dominant species (*q *= 2). Beta diversity is derived from the value 1−C_*qN,*_ where C_*qN*_ represents multi-assemblage similarities in effective numbers of species for *N* communities. The value of parameter q determines the weight given to relative species abundances in each measure. When *q *= 0, C_0_*N* represents the multi-assemblage similarity in effective species richness/numbers of observed species; when *q *= 1, C_1_*N* gives the multi-assemblage similarity in the effective number of common species; when *q *= 2, C_2_*N* gives the multi-assemblage similarity in the effective number of very abundant/dominant species. Error bars depict standard errors of each parameter obtained from bootstrapping at 1000 randomized iterations of the original data.

In oil palm, beta diversity at the largest grain, that is, grain 3, was highest when considering dominant species (^2^*β*_grain3_ = 0.77, SE = 0.005), relative to common species (^1^*β*_grain3_ = 0.49, SE = 0.006), and species identities regardless of relative abundance (^0^*β*_grain3_ = 0.27, SE = 0.023). The same pattern was observed across all grains, when beta diversity in oil palm was always highest for dominant species (*q* = 2) (Fig.3). Beta diversity of dominant species (^2^*β*) was significantly (*P* < 0.03) lower at the second grain (^2^*β*_grain2_ = 0.75, SE = 0.005) as compared to the first (^2^*β*_grain1_ = 0.77, SE = 0.004; ^2^*β*_grain1_ – ^2^*β*_grain2_ =0.015, *z* = −2.29, *P *< 0.03) and third grains, respectively, but ^2^*β*_grain1_ and ^2^*β*_grain3_ were not significantly different (^2^*β*_grain3_ = 0.77, SE = 0.005; ^2^*β*_grain1_ – ^2^*β*_grain3_ = −0.002, *z* = 0.37, *P* = 0.722).

When considering common species in oil palm (*q* = 1), beta diversity was overall highest at the largest grain. ^1^*β*_grain1_ was significantly lowest relative to ^1^*β*_grain2_ (^1^*β*_grain1_*−*^1^*β*_grain2_* *= −0.025, *z* = 3.38, *P* < 0.01) and ^1^*β*_grain3_ (^1^*β*_grain1_*−*^1^*β*_grain3_* = *−0.085, *z* = 10.96, *P* < 0.01), respectively. ^1^*β*_grain3_ was also higher than ^1^*β*_grain2_ (^1^*β*_grain3_*−*^1^*β*_grain2_* *= 0.06, *z* = 7.23, *P* < 0.01). When relative species abundances were not considered (*q* = 0), no significant differences were found between ^0^*β*_grain1_ and ^0^*β*_grain2,_^0^*β*_grain2_ and ^0^*β*_grain3._^0^*β*_grain3_ was, however, significantly higher than ^0^*β*_grain1_ (^0^*β*_grain3_*−*^0^*β*_grain1_* = *0.078, *z* = 2.83, *P* < 0.01). The patterns of change in beta diversity with grain in oil palm remained consistent when datasets were re-analyzed with singletons omitted.

As with oil palm, beta diversity among forest communities was also highest across all grains in terms of dominant species (*q* = 2), but lowest when species relative abundances were disregarded (*q* = 0), that is, ^2^*β* > ^1^*β* > ^0^*β* (Fig.3). In contrast to oil palm communities, however, ^2^*β* progressively declined with increasing grain. ^2^*β* was lowest at the third grain (^2^*β*_grain3_ = 0.30, SE = 0.011), and significantly (*P *< 0.05) lower than at the first (^2^*β*_grain1_ = 0.57, SE = 0.006; ^2^*β*_grain1_*−*^2^*β*_grain3_ = 0.268, *z* = −21.83, *P* < 0.01) and second (^2^*β*_grain2_ = 0.55, SE = 0.007; ^2^*β*_grain2_*−*^2^*β*_grain3_ = 0.249, *z* = −19.24, *P *< 0.01) grains, respectively. ^2^*β*_grain2_ was lower but not significantly different from ^2^*β*_grain1_ (^2^*β*_grain1_*−*^2^*β*_grain2_ = 0.018, *z* = −1.97, *P* = 0.05).

Beta diversity of common forest species (^1^*β*) changed more irregularly with increasing grain, but in general, ^1^*β* was highest at the second grain (^1^*β*_grain2_ = 0.34, SE = 0.005). ^1^*β*_grain3_ was lower than ^1^*β*_grain2,_ but this difference was not significant (^1^*β*_grain3_ = 0.33, SE = 0.006; ^1^*β*_grain2_*−*^1^*β*_grain3_ = 0.013, *z* = −1.71, *P* = 0.09). ^1^*β*_grain1_ was also not significantly lower than^1^*β*_grain3_ (^1^*β*_grain1_ = 0.32, SE = 0.004; ^1^*β*_grain1_*−*^1^*β*_grain3_ =, *z* = −0.001, *P* = 0.84). ^1^*β*_grain2_ was, nevertheless, significantly greater than ^1^*β*_grain1_ (^1^*β*_grain1_*−*^1^*β*_grain2_* *= −0.014, *z* = 2.28, *P* < 0.03). When relative species abundances were disregarded (*q* = 0), beta diversity (^0^*β*) generally increased with increasing grain – ^0^*β* was highest at the third and largest grain (^0^*β*_grain3_ = 0.32, SE = 0.018). ^0^*β* significantly (*P* < 0.017) increased from ^0^*β*_grain1_ (^0^*β*_grain1_ = 0.23, SE = 0.012) to ^0^*β*_grain3_ (^0^*β*_grain1_*−*^0^*β*_grain3_ = −0.085, *z* = 3.99, *P *< 0.01), and also from ^0^*β*_grain1_ to ^0^*β*_grain3_ (^0^*β*_grain2_ = 0.28, SE = 0.014; ^0^*β*_grain1_*−*^0^*β*_grain2_ = −0.046, *z* = 2.51, *P *< 0.05). When singletons were omitted from the dataset, the difference between ^0^*β*_grain1_ and ^0^*β*_grain2_ became nonsignificant (*P* > 0.05).

### Comparing beta diversities between oil palm and forest at different grains

Beta diversity when relative species abundances were disregarded (^0^*β*) was significantly (*P *< 0.05) higher in forest as compared to oil palm at the first (^0^*β*_oilpalm1_*−*^0^*β*_forest1_ = −0.05, *z* = 2.54, *P* = 0.01) and second (^0^*β*_oilpalm2_*−*^0^*β*_forest2_ = −0.06, *z* = 2.63, *P* = 0.01) grains, respectively. At the largest grain, the difference between ^0^*β*_oilpalm3_ and ^0^*β*_forest3_ was not significant (^0^*β*_oilpalm3_*−*^0^*β*_forest3_ = −0.05, *z* = 1.84, *P* = 0.07). Beta diversity of common species was significantly (*P* < 0.05) greater in oil palm than in forest across all three grains (^1^*β*_oilpalm1_*−*^1^*β*_forest1_ = 0.07; ^1^*β*_oilpalm2_*−*^1^*β*_forest2_ = 0.09; ^1^*β*_oilpalm3_*−*^1^*β*_forest3_ = 0.16) (Fig.3). Beta diversity of dominant species was also significantly (*P* < 0.05) higher in oil palm than forest for all grains (^2^*β*_oilpalm1_*−*^2^*β*_forest1_ = 0.20; ^2^*β*_oilpalm2_*−*^2^*β*_forest2_ = 0.21; ^2^*β*_oilpalm3_*−*^2^*β*_forest3_* *= 0.47) (Fig.3). When data were re-analyzed without singletons, most trends remained the same, except ^0^*β* was no longer significantly higher in forest than oil palm at the first two sampling grains.

## Discussion

We found, using our multigrain hierarchical sampling scheme, that ground-foraging ant communities in oil palm are more heterogeneous than usually assumed of monocultures, especially in comparison with forest communities. This heterogeneity becomes more evident with respect to relatively abundant species and at larger sample sizes. As expected, forest communities had more ant species at every grain, that is, higher alpha diversity. But there were two unexpected and important results. First, the average percentage similarity between oil palm and forest ant communities generally increased with increasing sampling grain (Fig.2); this is unexpected if we view oil palm as a homogeneous monoculture, supporting a similar set of species at every grain. Second, the beta diversities of common and very abundant (dominant) species were always higher among oil palm communities, relative to forest communities, regardless of grain (Fig.3). These results suggest that studies which overlook beta diversity and sampling grain effects may distort the true impacts of conversion of natural habitats to agriculture on biodiversity in modified landscapes.

### Average similarity between oil palm and forest communities

The increase in similarity between oil palm and forest communities with sampling grain raises the question of how a “forest species” should be operationally defined. This increase in similarity may be attributed to the higher probability of obtaining shared species when larger sampling grains in oil palm generate bigger species pools. This implies that oil palm communities are heterogeneous, but this heterogeneity is only captured in large samples. The accumulation of diversity with increasing sample size is unexpected for oil palm, as intensive agriculture is usually thought to homogenize communities over large spatial scales (Karp et al. 2012). In addition, the shifts in relative species abundances with grain may also contribute to increased average similarities of common and dominant species (Fig.2). These results suggest that species may be erroneously assumed to be restricted to forest simply because they do not, or rarely, occur in small samples from oil palm. The species *Tetramorium smithi* (Mayr, 1879), for example, found in both oil palm and forest (Table S1), entered the top abundance ranks only at larger grains (Appendix S2). Furthermore, our results are not confounded by potential spillover effects, as samples were collected well away from plantation-forest boundaries. In addition, we can be confident that rare species occurrence indicates the existence of actual ant colonies inhabiting the area being sampled, as ants are relatively sessile organisms with restricted home ranges (Alonso 2000). While total diversity in agricultural landscapes is undoubtedly lower relative to native forest landscapes, there may potentially be more species of conservation importance in agriculture than assumed, but these may only be captured if samples are sufficiently large.

### Beta diversity of ground-foraging ant communities in oil palm and forest

#### Beta diversity of species identities

Beta diversity of species identities was generally higher in forest than in oil palm, but comparisons were sensitive to both grain size and the inclusion of singletons. This indicates that beta diversity of species identities in oil palm may be comparable to those in forest, but only at sufficiently large sampling grains. Upon excluding singletons, beta diversities in oil palm and forest were not significantly different at every grain, suggesting that forest communities are more heterogeneous than those in oil palm at smaller grains mainly due to the occurrence of rare species, when relative species abundances are disregarded. Rare species, however, do not significantly enhance forest beta diversity relative to oil palm beta diversity at the largest grain. Consequently, these results entail the fact that heterogeneity among ant communities in oil palm tends to increase at larger scales, alongside the probability of obtaining rare species. Assessing small samples may therefore underestimate the actual faunal diversity in oil palm and misrepresent the true conservation value of the entire tropical agricultural landscape.

#### Beta diversity of common and dominant species

Unexpectedly, the beta diversities of common and dominant ant communities were strikingly higher in oil palm than in forest at all sampling grains: the differences were particularly marked for dominant species, and at the largest grain (Fig.3). For forest ant communities, this could be explained by greater habitat stability over time, promoting the persistence of historically established species ranges and favouring homogenization among discrete communities (Juen and De Marco 2011). The possibility of historic events of range expansion imprinting on and homogenizing forest communities is compounded by the tendency of ant colonies to persist once established in a particular location, provided local environmental conditions do not undergo sudden drastic changes that displace entire assemblages, that is, agricultural conversion (Andersen et al. 2012). Low beta diversity of common and dominant species at the largest grain may be attributed to the inclusion of widely dispersed species which qualify as relatively more abundant only in larger grains. This is shown by the rise in abundance rank of certain forest species with increasing grain, such as *Lophomyrmex bedoti* (Emery, 1893) and *Leptogenys diminuta* (Smith, F., 1857) (Appendix S2).

In contrast to old growth forest, the younger oil palm environment is much more dynamic and less stable. Local microclimate conditions change as oil palm crops grow (Luskin and Potts 2011), giving rise to the potential for supporting temporally disparate communities (Foster et al. 2011). The variation in stand age of blocks within the oil palm matrix may thus enable the existence of more heterogeneous ant communities than expected of a typical monoculture. Furthermore, variable immigration and historic events of community assembly within separate oil palm blocks following conversion may also boost heterogeneity among communities of common and dominant species in oil palm at the largest grain (Chase 2003; Fukami 2010). The relatively lower beta diversity of dominant species for oil palm at the second grain (Fig.3) implies that spatially discrete plantations may support similar dominant species, as each sampling unit usually comprises pooled samples from the same plantation. Dominant species may share identical traits that allow them to exploit the relatively homogeneous conditions of each oil palm site soon after conversion, quickly achieving numerical dominance and modifying resources in situ that limit the immigration of other species in later stages of community assembly (Fukami 2010). Surrounding species source pools providing immigrants to these oil palm sites may be very different in composition at the largest grain, increasing the variability of species arriving first and establishing dominance. As a result, beta diversities of common and dominant oil palm species become highest at the largest grain.

## Conclusion

Overall, our results show that the ground-foraging ant communities in tropical oil palm are not as homogeneous, in comparison with forest communities, as is often expected for fauna in large-scale monocultures. As shown in previous studies, homogenization or differentiation of communities in fragmented and heavily modified landscapes is ultimately contingent on spatial configuration of the landscape, and the varying successional pathways undergone by each discrete community (Arroyo-Rodríguez et al. 2013). Furthermore, beta diversity in oil palm seems to increase with rising sampling grain, suggesting that heterogeneity among oil palm communities becomes more evident at larger sample sizes. Therefore, studies which overlook beta diversity and are based solely on small samples probably misinterpret the impacts of agricultural conversion on biodiversity. While we do not dispute the fact that the preservation of pristine tropical forest should remain a conservation priority, our results also show that there is also substantial diversity present in large-scale agriculture which should not be ignored.

We acknowledge that many oil palm species may be recognized as wide-ranging tramp or exotic species of little conservation interest, such as *Monomorium floricola* and *Anoplolepis gracilipes*. Such species, however, may still play important roles in maintaining ecosystem function, especially if naturalized within their respective communities (Prévot-Julliard et al. 2011). Besides the preservation of rare species, the maintenance of sustainable ecosystems should also be a major conservation objective, especially for fragmented landscapes with multiple land-use types (Tscharntke et al. 2005).

The effective mitigation of large-scale impacts of forest conversion on tropical biodiversity has to include the maintenance of diversity in landscapes dominated by agriculture. The understanding of beta diversity, and its underlying processes, at multiple scales is therefore necessary, for the management and sustenance of biodiversity present in modified agricultural landscapes.

## Supporting Information

**Appendix S1.** Sampling completeness and coverage curves.

**Appendix S2.** Changes in species ranked abundance (biomass/mg) with sampling grain.

**Appendix S3.** Ranked species abundance distribution curves.

**Table S1.** Total species list.
