# Supplementary material for: The effects of forest conversion to oil palm on ground-foraging ant communities depend on beta diversity and sampling grain
Source: Ecol Evol. 2015 Jul 14;5(15):3159–70. doi: 10.1002/ece3.1592 (PMC4559058; doi:10.1002/ece3.1592)
Supplement: Supplementary file 1 [file ece30005-3159-sd1.docx]

**Appendix S1** *Sampling completeness and coverage curves*

Curves assessing sampling completeness and coverage for 26 oil palm and 21 forest plots – each sample was obtained from a single 100 m^2^ plot. Extrapolated values are represented by dashed lines. Only observed species richness ($D_{\alpha}^{0})$ was used to plot the curves. The 95% confidence intervals (shaded area surrounding each curve) were obtained from a bootstrap method based on 100 replications of the reference sample set.
