# Supplementary material for: The effects of forest conversion to oil palm on ground-foraging ant communities depend on beta diversity and sampling grain
Source: Ecol Evol. 2015 Jul 14;5(15):3159–70. doi: 10.1002/ece3.1592 (PMC4559058; doi:10.1002/ece3.1592)
Supplement: Supplementary file 2 [file ece30005-3159-sd2.docx]

**Appendix S2** *Changes in species ranked abundance (biomass) with sampling grain*

Bar graphs depicting the top 20 (**A**) oil palm, and (**B**) forest species ranked in terms of abundance (LOG_10_ of Mean biomass (mg) per sampled grain where the species occurred) across three different sampling grains(1 – 3: smallest – largest) (**A**) Colourless bars indicate species from oil palm that are also top-ranked from forest samples. (**B**) Grey bars highlight forest species that remained in the top 10 across all 3 grains. Colourless bars indicate two species, *Leptogenys diminuta* and *Lophomyrmex bedoti*, which moved up the rankings progressively with each level of increase in grain.
