# Supplementary material for: The effects of forest conversion to oil palm on ground-foraging ant communities depend on beta diversity and sampling grain
Source: Ecol Evol. 2015 Jul 14;5(15):3159–70. doi: 10.1002/ece3.1592 (PMC4559058; doi:10.1002/ece3.1592)
Supplement: Supplementary file 3 [file ece30005-3159-sd3.docx]

**Appendix S3** *Ranked species abundance distribution curves*

Species abundance curves for ground-foraging ant species in oil palm and forest, ranked in terms of the mean biomass (mg) per sample the species was found. Biomass serves as a surrogate for individual counts. Each sample here comprises one unit of the smallest sampling grain.
