# Supplementary material for: The effects of forest conversion to oil palm on ground-foraging ant communities depend on beta diversity and sampling grain
Source: Ecol Evol. 2015 Jul 14;5(15):3159–70. doi: 10.1002/ece3.1592 (PMC4559058; doi:10.1002/ece3.1592)
Supplement: Supplementary file 4 [file ece30005-3159-sd4.docx]

**Table S1** *Total species list:* List of 223 ant morphospecies obtained from 26 oil palm and 21 natural forest sites of the smallest sampling grain (100 m^2^). Species codes with the abbreviation “SKY” indicate specimens that have been matched with those in the personal reference collection of Seiki Yamane (Kagoshima University), whereas codes with the abbreviation “EG” mark specimens matched with those in the personal reference collection of Katsuyuki Eguchi (Tokyo Metropolitan University).

| Subfamily | Genus | No.of species | Morphospecies | Forest | Oil Palm |
| --- | --- | --- | --- | --- | --- |
| *Aenictinae* | *Aenictus* | 3 | *Aenictus* 2 | 1 |  |
|  |  |  | *Aenictus* 3*(Ceylonicus*.sp.B) | 1 |  |
|  |  |  | *Aenictus* 4*(Ceylonicus.*sp.A) | 1 |  |
| *Ambyloponinae* | *Prionopelta* | 1 | *Prionopelta* 1 | 1 |  |
| *Cerapachyinae* | *Cerapachys* | 2 | *Cerapachys*1(sp.18.of.SKY) | 1 |  |
|  |  |  | *Cerapachys* 2(sp.23.of.SKY) | 1 |  |
| *Dolichoderinae* | *Bothriomyrmex* | 1 | *Bothriomyrmex* 1(sp.1.of.SKY) |  | 1 |
|  | *Dolichoderus* | 5 | *Dolichoderus* 1 | 1 |  |
|  |  |  | *Dolichoderus* 2 | 1 |  |
|  |  |  | *Dolichoderus* 3 | 1 |  |
|  |  |  | *Dolichoderus cuspivatus* | 1 |  |
|  |  |  | *Dolichoderus thoracicus* | 1 |  |
|  | *Loweriella* | 1 | *Loweriella boltoni* | 1 |  |
|  | *Philidris* | 1 | *Philidris* 1(sp.1.of.SKY) | 1 |  |
|  | *Tapinoma* | 2 | *Tapinoma melanocephalum* | 1 | 1 |
|  |  |  | *Tapinoma* 2 |  | 1 |
|  | *Technomyrmex* | 5 | *Technomyrmex pratensis* | 1 |  |
|  |  |  | *Technomyrmex* 1 | 1 | 1 |
|  |  |  | *Technomyrmex albipes* | 1 |  |
|  |  |  | *Technomyrmex kraepelini* | 1 | 1 |
|  |  |  | *Technomyrmex modiglianii* | 1 |  |
| *Dorylinae* | *Dorylus* | 1 | *Dorylus laevigatus* |  | 1 |
| *Ectatomminae* | *Gnamptogenys* | 4 | *Gnamptogenys* 1(sp.5.of.SKY) | 1 | 1 |
|  |  |  | *Gnamptogenys* 2(sp.9.of.SKY) | 1 |  |
|  |  |  | *Gnamptogenys posteropsis* | 1 |  |
|  |  |  | *Gnamptogenys* 4(cf.*C.binghamii*) | 1 |  |
| *Formicinae* | *Anoplolepis* | 1 | *Anoplolepis gracilipes* |  | 1 |
|  | *Camponotus* | 8 | *Camponotus*(sp.9.of.SKY.*Colobopris*) | 1 |  |
|  |  |  | *Camponotus* 2(sp.48.of.SKY.*Tanaemyrmex*) | 1 |  |
|  |  |  | *Camponotus arrogans* | 1 | 1 |
|  |  |  | *Camponotus* 3(sp.15.of.SKY.*Tanaemyrmex*) | 1 |  |
|  |  |  | *Camponotus gigas* | 1 |  |
|  |  |  | *Camponotus* 5 | 1 |  |
|  |  |  | *Camponotus* 6*(Camponotus.*cf.*C.saundersi)* | 1 |  |
|  |  |  | *Camponotus* 9(sp.38.of.SKY.*Colobopsis*) |  | 1 |
|  | *Euprenolepis* | 4 | *Euprenolepis thrix* | 1 | 1 |
|  |  |  | *Euprenolepis wittei* | 1 |  |
|  |  |  | *Euprenolepis variegata* | 1 |  |
|  |  |  | *Euprenolepis procera* | 1 |  |
|  | *Nylanderia* | 10 | *Nylanderia* 1(sp.5.of.SKY) |  | 1 |
|  |  |  | *Nylanderia* 1i |  | 1 |
|  |  |  | *Nylanderia 2*(cf.sp.5.of.SKY) | 1 | 1 |
|  |  |  | *Nylanderia* 2i(sp.6.of.SKY) | 1 |  |
|  |  |  | *Nylanderia* 2ii(*Nylanderia*.sp.unk) | 1 |  |
|  |  |  | *Nylanderia* 2iii*(*sp.23.of.SKY) | 1 |  |
|  |  |  | *Nylanderia* 3 | 1 |  |
|  |  |  | *Nylanderia* 4(sp.18.of.SKY) | 1 |  |
|  |  |  | *Nylanderia* 4i(sp.19.of.SKY) | 1 |  |
|  |  |  | *Nylanderia* 5(sp.28.of.SKY) | 1 |  |
|  | *Oecophylla* | 1 | *Oecophylla smaragdina* |  | 1 |
|  | *Paraparatrechina* | 3 | *Paraparatrechina*.sp.8.of.SKY |  | 1 |
|  |  |  | *Paraparatrechina 1*(sp.7.of.SKY) | 1 | 1 |
|  |  |  | *Paraparatrechina opaca* | 1 | 1 |
|  | *Paratrechina* | 1 | *Paratrechina longicornis* |  | 1 |
|  | *Plagiolepis* | 1 | *Plagiolepis* 1(sp.1.of.SKY) | 1 | 1 |
|  | *Polyrachis* | 2 | *Polyrachis* 1 | 1 |  |
|  |  |  | *Polyrachis* 2 | 1 |  |
|  | *Prenolepis* | 1 | *Prenolepis* 1 | 1 |  |
|  | *Pseudolasius* | 2 | *Pseudolasius* 2 | 1 |  |
|  |  |  | *Pseudolasius* 3(sp.3.of.SKY) | 1 |  |
|  | *Myrmoteras* | 1 | *Myrmoteras*.sp.14.of.SKY | 1 |  |

Table S1 continued

| Subfamily | Genus | No.of species | Morphospecies | Forest | Oil Palm |
| --- | --- | --- | --- | --- | --- |
| *Myrmicinae* | *Acanthomyrmex* | 2 | *Acanthomyrmex* 1 | 1 |  |
|  |  |  | *Acanthomyrmex* 2 | 1 |  |
|  | *Calyptomyrmex* | 1 | *Calyptomyrmex* 1(near *C.loweryi*) |  | 1 |
|  | *Cardiocondyla* | 2 | *Cardiocondyla tjibodana* |  | 1 |
|  |  |  | *Cardiocondyla wroughtonii* | 1 |  |
|  | *Crematogaster* | 12 | *Crematogaster coriaria.Paracrema* |  | 1 |
|  |  |  | *Crematogaster* 10(cf.sp.134.of.SKY.*Orthocrema*) | 1 |  |
|  |  |  | *Crematogaster* 11 | 1 |  |
|  |  |  | *Crematogaster* 12(sp.61.of.SKY) | 1 | 1 |
|  |  |  | *Crematogaster* 13(cf.*C.baduri*) | 1 |  |
|  |  |  | *Crematogaster* 14 |  | 1 |
|  |  |  | *Crematogaster biroi.bandarensis.Orthocrema* | 1 | 1 |
|  |  |  | *Crematogaster longipilosa.Orthocrema* |  | 1 |
|  |  |  | *Crematogaster rogenhoferi.Crematogaster* |  | 1 |
|  |  |  | *Crematogaster* 7*(Crematogaster*.sp.) | 1 |  |
|  |  |  | *Crematogaster fraxatrix.Crematogaster* | 1 |  |
|  |  |  | *Crematogaste r*9*(*cf*.C.modiglianii)* | 1 |  |
|  | *Gauromyrmex* | 1 | *Gauromyrmex* 1 | 1 |  |
|  | *Lophomyrmex* | 2 | *Lophomyrmex bedoti* | 1 | 1 |
|  |  |  | *Lophomyrmex longicornis* | 1 | 1 |
|  | *Mayriella* | 1 | *Mayriella transfuga* | 1 |  |
|  | *Meranoplus* | 1 | *Meranoplus mucronatus* | 1 |  |
|  | *Monomorium* | 6 | *Monomorium destructor* | 1 | 1 |
|  |  |  | *Monomorium floricola* | 1 | 1 |
|  |  |  | *Monomorium* 3 | 1 | 1 |
|  |  |  | *Monomorium* 4(sp.1.of.SKY) | 1 | 1 |
|  |  |  | *Monomorium* 5(sp.5.of.SKY) | 1 |  |
|  |  |  | *Monomorium talpa* | 1 |  |
|  | *Myrmecina* | 3 | *Myrmecina* 1 | 1 | 1 |
|  |  |  | *Myrmecina spinosa* | 1 | 1 |
|  |  |  | *Myrmecina 2* | 1 |  |
|  | *Myrmicaria* | 4 | *Myrmicaria adpressipilosa* | 1 |  |
|  |  |  | *Myrmicaria* 3 | 1 |  |
|  |  |  | *Myrmicaria* 3i | 1 |  |
|  |  |  |  |  |  |
|  | *Oligomyrmex* | 6 | *Oligomyrmex*.sp.2.of.SKY | 1 | 1 |
|  |  |  | *Oligomyrmex.*sp.3.of.SKY | 1 |  |
|  |  |  | *Oligomyrmex* 2(sp.18.of.SKY) | 1 | 1 |
|  |  |  | *Oligomyrmex* 4(sp.13.of.SKY) | 1 |  |
|  |  |  | *Oligomyrmex* 5(cf.sp.22.of.SKY) |  | 1 |
|  |  |  | *Oligomyrmex* 7(cf.sp.5.of.SKY) |  | 1 |
|  | *Pheidole* | 35 | *Pheidole lucioccipitalis* | 1 |  |
|  |  |  | *Pheidole* *comata/longipes* |  | 1 |
|  |  |  | *Pheidole fervens* | 1 | 1 |
|  |  |  | *Pheidole* 12 | 1 | 1 |
|  |  |  | *Pheidole aristotelis* | 1 |  |
|  |  |  | *Pheidole* 14 | 1 |  |
|  |  |  | *Pheidole* 15 | 1 | 1 |
|  |  |  | *Pheidole tjibodana* | 1 |  |
|  |  |  | *Pheidole poringensis* | 1 |  |
|  |  |  | *Pheidole* 18 | 1 |  |
|  |  |  | *Pheidole quadrensis* | 1 |  |
|  |  |  | *Pheidole aglae* | 1 | 1 |
|  |  |  | *Pheidole* 22*(near P.rabo)* | 1 |  |
|  |  |  | *Pheidole* 23 | 1 |  |
|  |  |  | *Pheidole* 24(cf.sp.25.of.SKY) | 1 |  |
|  |  |  | *Pheidole sabahna* | 1 |  |
|  |  |  | *Pheidole parvicorpus* | 1 |  |
|  |  |  | *Pheidole rabo* | 1 |  |
|  |  |  | *Pheidole fantasia* | 1 |  |
|  |  |  | *Pheidole plagiaria* | 1 | 1 |
|  |  |  | *Pheidole* 30(cf.*P.quadrensi*s) | 1 |  |
|  |  |  | *Pheidole* 31 | 1 |  |
|  |  |  | *Pheidole gombakensis* | 1 |  |
|  |  |  |  |  |  |

Table S1 continued

| Subfamily | Genus | No.of species | Morphospecies | Forest | Oil Palm |
| --- | --- | --- | --- | --- | --- |
|  |  |  | *Pheidole* 33(cf.*P.tjibodana*) | 1 |  |
|  |  |  | *Pheidole cariniceps* | 1 | 1 |
|  |  |  | *Pheidole* 4i(near *P.annexus*) | 1 | 1 |
|  |  |  | *Pheidole sarawakana* | 1 | 1 |
|  |  |  | *Pheidole angulicollis* | 1 |  |
|  |  |  | *Pheidole clypeocornis* | 1 | 1 |
|  |  |  | *Pheidole* 7i |  | 1 |
|  |  |  | *Pheidole* 7iii(sp.EG.30.aff.*P.hortensis*) | 1 | 1 |
|  |  |  | *Pheidole rugifera* | 1 | 1 |
|  |  |  | *Pheidole quadricuspis.*Emery | 1 |  |
|  |  |  | *Pheidole pheidolacanthiuus* (sp.EG.77) | 1 |  |
|  |  |  | *Pheidole* 9ii(cf.*P.spinicornis*) | 1 |  |
|  | *Pheidologeton* | 4 | *Pheidologeton pygmaeus* | 1 |  |
|  |  |  | *Pheidologeton.*unknown sp. | 1 | 1 |
|  |  |  | *Pheidologeton* 1*(P.affinis.*sp.5.of.SKY) | 1 | 1 |
|  |  |  | *Pheidologeton silenus* | 1 | 1 |
|  | *Pristomyrmex* | 1 | *Pristomyrmex* 1 |  | 1 |
|  | *Proatta* | 1 | *Proatta* 1 | 1 |  |
|  | *Pyramica* | 4 | *Pyramica mitis* | 1 |  |
|  |  |  | *Pyramica* 1 |  | 1 |
|  |  |  | *Pyramica karawajewi* |  | 1 |
|  |  |  | *Pyramica jacobsoni* | 1 | 1 |
|  | *Recurvidris* | 3 | *Recurvidris* 1(sp.2.of.SKY) | 1 |  |
|  |  |  | *Recurvidris browni* | 1 |  |
|  |  |  | *Recurvidris kemneri* | 1 | 1 |
|  | *Rhopalomastix* | 1 | *Rhopalomastix*(cf.sp.3.of.SKY) | 1 |  |
|  | *Rhoptromyrmex* | 1 | *Rhoptromyrmex wroughtonii* | 1 | 1 |
|  | *Solenopsis* | 3 | *Solenopsis*.sp.15.of.SKY | 1 | 1 |
|  |  |  | *Solenopsis*.sp.9.of.SKY | 1 |  |
|  |  |  | *Solenopsis* 1 | 1 | 1 |
|  | *Strumigenys* | 7 | *Strumigenys.*unknown sp. | 1 |  |
|  |  |  | *Strumigenys arrogantia* |  | 1 |
|  |  |  | *Strumigenys* 2 | 1 |  |
|  |  |  | *Strumigenys signaea* | 1 |  |
|  |  |  | *Strumigenys godeffroyi* |  | 1 |
|  |  |  | *Strumigenys koningsbergeri* | 1 |  |
|  |  |  | *Strumigenys rofocara* | 1 | 1 |
|  | *Tetramorium* | 21 | *Tetramorium pacificum* |  | 1 |
|  |  |  | *Tetramorium*(close to *T.brevidentatum*) | 1 |  |
|  |  |  | *Tetramorium.*unknown sp. | 1 |  |
|  |  |  | *Tetramorium1*(cf.*T.tonganum*) | 1 | 1 |
|  |  |  | *Tetramorium meshena* | 1 | 1 |
|  |  |  | *Tetramorium* 13 | 1 | 1 |
|  |  |  | *Tetramorium* 14(near *T.rugigaster*) | 1 |  |
|  |  |  | *Tetramorium simillimum* |  | 1 |
|  |  |  | *Tetramorium* 1i(cf.*T.aptum*) |  | 1 |
|  |  |  | *Tetramorium noratum* | 1 | 1 |
|  |  |  | *Tetramorium scabrum* | 1 | 1 |
|  |  |  | *Tetramorium eleates* |  | 1 |
|  |  |  | *Tetramorium* 4(sp.1.of.SKY.cf.*T.adpressum*) | 1 | 1 |
|  |  |  | *Tetramorium kheperra* |  | 1 |
|  |  |  | *Tetramorium inglebyi* |  | 1 |
|  |  |  | *Tetramorium aptum* | 1 | 1 |
|  |  |  | *Tetramorium* 6i(cf.*T.aptum*) | 1 | 1 |
|  |  |  | *Tetramorium bicarinatum* |  | 1 |
|  |  |  | *Tetramorium smithi* | 1 | 1 |
|  |  |  | *Tetramorium lanuginosum* |  | 1 |
|  |  |  | *Tetramorium* 9i(cf.*T.parvum*) | 1 | 1 |
|  | *Vollenhovia* | 5 | *Vollenhovia* 1 |  | 1 |
|  |  |  | *Vollenhovia* 2*(*close to *V.fridae)* | 1 | 1 |
|  |  |  | *Vollenhovia* 3 |  | 1 |
|  |  |  | *Vollenhovia* 4 | 1 | 1 |
|  |  |  | *Vollenhovia* 5 |  | 1 |
|  |  |  |  |  |  |

Table S1 continued

| Subfamily | Genus | No.of species | Morphospecies | Forest | Oil Palm |
| --- | --- | --- | --- | --- | --- |
| Ponerinae | *Anochetus* | 3 | *Anochetus* 1 | 1 |  |
|  |  |  | *Anochetus* 2*(A.graeffei.*complex) |  | 1 |
|  |  |  | *Anochetus* 3(sp.4.of.SKY) | 1 |  |
|  | *Cryptopone* | 1 | *Cryptopone* 1 | 1 | 1 |
|  | *Diacamma* | 2 | *Diacamma* 1 | 1 |  |
|  |  |  | *Diacamma* 2 |  | 1 |
|  | *Hypoponera* | 5 | *Hypoponera*(cf.sp.5.of.SKY) | 1 |  |
|  |  |  | *Hypoponera* 1(sp.15.of.SKY) | 1 |  |
|  |  |  | *Hypoponera* 2(cf.sp.40.of.SKY) | 1 | 1 |
|  |  |  | *Hypoponera* 3i(cf.sp.19.of.SKY) | 1 |  |
|  |  |  | *Hypoponera* 3(sp.19.of.SKY) |  | 1 |
|  | *Leptogenys* | 9 | *Leptogenys mutabilis* | 1 | 1 |
|  |  |  | *Leptogenys* 10 (unknown sp.) | 1 |  |
|  |  |  | *Leptogenys* 11(unknown sp.) | 1 |  |
|  |  |  | *Leptogenys diminuta* | 1 | 1 |
|  |  |  | *Leptogenys borneensis* | 1 |  |
|  |  |  | *Leptogenys myops* | 1 |  |
|  |  |  | *Leptogenys peugueti* | 1 | 1 |
|  |  |  | *Leptogenys* 8(near *L.mutabilis*.sp.6.of.SKY) | 1 |  |
|  |  |  | *Leptogenys paruula* | 1 |  |
|  | *Odontomachus* | 1 | *Odontomachus* 1*(O.rixosus*-complex) | 1 |  |
|  | *Odontoponera* | 2 | *Odontoponera denticulata* |  | 1 |
|  |  |  | *Odontoponera transversa* | 1 | 1 |
|  | *Pachycondyla* | 8 | *Pachycondyla amblyops* | 1 |  |
|  |  |  | *Pachycondyla*(sp.1.of.SKY.*Brachyponera*) | 1 | 1 |
|  |  |  | Pachycondyla(sp.28.of.SKY.*Brachyponera*) | 1 | 1 |
|  |  |  | *Pachycondyla sharpi.Trachymesops)* | 1 | 1 |
|  |  |  | *Pachycondyla leeuwenhoeki.Ectomomyrmex* | 1 |  |
|  |  |  | *Pachycondyla* 4(sp.13.of.SKY.*Mesoponera*) | 1 |  |
|  |  |  | *Pachycondyla pilidorsalis*.*Brachyponera* | 1 |  |
|  |  |  | *Pachycondyla7*(cf.sp.3.of.SKY) | 1 |  |
|  | *Platythyrea* | 2 | *Platythyrea parallela* |  | 1 |
|  |  |  | *Platythyrea tricuspidatus* |  | 1 |
|  | *Ponera* | 1 | *Ponera* 6(sp.2.of.SKY) | 1 |  |
| Pseudomyrmecinae | *Tetraponera* | 1 | *Tetraponera* 1 | 1 |  |
|  |  |  |  |  |  |
|  |  |  | Total number of species | 181 | 105 |
|  |  |  |  |  |  |

**Table S1.1** Recent taxonomic name changes and synonymizations. SKY – Seiki Yamane’s Reference Collection

| **ID at time of study** | **Changed Names/ Synonymizations** | **Notes** |
| --- | --- | --- |
| *Bothriomyrmex* sp 1. of SKY | Junior synonym of *Bothriomyrmex* – *Chronoxenus* – raised to genus (Dubovikoff, 2005). | Here we choose to respect and support Prof. Seiki Yamane’s identification of the genus at the time of this study |
| *Oligomyrmex* spp. | *Carebara* spp. |  |
| *Pheidologeton* spp. | *Carebara* spp. |  |
| *Pyramica* spp. | *Strumigenys* spp. |  |
| *Rhoptromyrmex wroughtonii* | *Tetramorium wroughtonii* |  |
| *Pachycondyla amblyops* | *Buniapone amblyops* |  |
| *Pachycondyla sharpi* | *Euponera sharpi* |  |
| *Pachycondyla leeuwenhoeki* | *Ectomyrmex leeuwenhoeki* |  |
| *Pachycondyla pilidorsalis* | *Brachyponera pilidorsalis* |  |
| *Pachycondyla* sp.1,3,13,28 of SKY |  | New taxonomic names unknown, as ID only up to morphospecies |
